# Group Dynamics and Landscape Features Constrain the Exploration of Herds in Fusion-Fission Societies: The Case of European Roe Deer

**DOI:** 10.1371/journal.pone.0034678

**Published:** 2012-03-30

**Authors:** Olivier Pays, Daniel Fortin, Jean Gassani, Jean Duchesne

**Affiliations:** 1 Groupe Ecologie et Conservation, Université d'Angers, Angers, France; 2 Centre de Recherche et Formation en Eco-Ethologie, Université de Reims, Boult-aux-Bois, France; 3 Comportement et Ecologie de la Faune Sauvage, Institut National de Recherche Agronomique, Castanet-Tolosan, France; 4 Département de biologie, Université Laval, Québec, Canada; 5 Université de Djibouti, Djibouti, République de Djibouti; 6 Unité de recherche Paysage, Agrocampus Ouest, Angers, France; University of Bristol, United Kingdom

## Abstract

Despite the large number of movement studies, the constraints that grouping imposes on movement decisions remain essentially unexplored, even for highly social species. Such constraints could be key, however, to understanding the dynamics and spatial organisation of species living in group fusion-fission systems. We investigated the winter movements (speed and diffusion coefficient) of groups of free-ranging roe deer (*Capreolus capreolus*), in an agricultural landscape characterised by a mosaic of food and foodless patches. Most groups were short-lived units that merged and split up frequently during the course of a day. Deer groups decreased their speed and diffusion rate in areas where food patches were abundant, as well as when travelling close to main roads and crest lines and far from forests. While accounting for these behavioural adjustments to habitat features, our study revealed some constraints imposed by group foraging: large groups reached the limit of their diffusion rate faster than small groups. The ability of individuals to move rapidly to new foraging locations following patch depression thus decreases with group size. Our results highlight the importance of considering both habitat heterogeneity and group dynamics when predicting the movements of individuals in group fusion-fission societies. Further, we provide empirical evidence that group cohesion can restrain movement and, therefore, the speed at which group members can explore their environment. When maintaining cohesion reduces foraging gains because of movement constraints, leaving the group may become a fitness-rewarding decision, especially when individuals can join other groups located nearby, which would tend to maintain highly dynamical group fusion-fission systems. Our findings also provide the basis for new hypotheses explaining a broad range of ecological patterns, such as the broader diet and longer residency time reported for larger herbivore groups.

## Introduction

Living in groups presents an individual with benefits, including a decrease in predation risk due to dilution effects and collective detection of predators, an opportunity to increase foraging time by decreasing its own scanning rate in response to the vigilance of others, and an opportunity to glean information on the location of high-quality food patches from the behaviour of competitors [1]–[6]. Group living also carries some well-known costs, such as the potential for aggression by conspecifics, an increase in resource competition and pseudo-interference, a risk of kleptoparasitism, and an increase in parasite burdens and disease transmission [7]–[16]. An additional, but less documented, cost of group living lies in the constraint imposed by group cohesion on the movements of individuals. Group cohesion can only exist if group members synchronise their movements. Considering groups as mobile units, they should become slower as their size increases because of the inertia that is generated by potentially conflicting path directions among an increasing number of group members. The capacity to explore landscapes, therefore, should vary as a function of group size.

The constraint imposed by group cohesion on movements can be critical for understanding the spatial organisation of social animals in landscapes because movement is one of the most fundamental mechanisms of animal distribution [17]. There has been a growing interest in understanding the interplay between movement decisions, resource use, and animal distribution in heterogeneous landscapes [18]–[21], [22 for reviews]. Despite the large number of existing movement studies, the effects of group dynamics on movement decisions have remained essentially unexplored, even for highly social species.

Various models of animal movements have been used to investigate a range of ecological themes such as dispersal, distribution and foraging strategies of terrestrial vertebrates [23]–[31]. A simple, yet common, approach to studying animal movement is to contrast observed paths with expectations for random walkers [32]–[37]. Random-walk models assume a succession of random steps, with the direction and length of each step being independent of previous steps and environmental features. The Euclidean distance (squared) between a starting location and the current location (i.e., net-squared displacement) should increase linearly over time for random walkers [38]. The asymptotic slope (specifically: 0.25× slope) of the relationship then corresponds to the diffusion coefficient [39], which indicates the long-term rate of spread that would be expected for a population of random walkers. The same relationship should apply to “correlated” random walkers when time is large [40]. A correlated random walk has the same properties as a random walk, except that the direction of a given step depends on the direction of the previous one. Consideration of inter-individual variations in the increase of net-squared displacement over time has proved to be useful in explaining temporal dynamics of animal population distributions [e.g., 35]. Likewise, the study of variations in diffusion coefficient in different landscapes or among groups of different sizes should reveal potential constraints on the spreading rate of individuals. For example, diffusion coefficient should decrease with group size if group cohesion constrains movement.

We investigated the winter movements of groups of free-ranging roe deer (*Capreolus capreolus*) during daylight hours in an extremely open agricultural landscape. As is the case for many large mammalian herbivores [e.g.], [ 41]–[47], roe deer groups are non-permanent units that often merge and split up during the day. Group dynamics of this deer population consist of a fusion-fission system, with a median group lifetime of several hours before one or several individuals join or leave the group [48]. Here we tested for the effect of both landscape (i.e., elevation, distance to anthropogenic landscape features, and distribution of food patches) and herd (i.e., group size) factors on group exploration capacity, as indicated by its movement speed and diffusion coefficient. Group exploration capacity was examined with these two descriptors (speed and diffusion rate) because groups could, for example, travel fast (high speed) while staying near their current location (low diffusion rate). The landscape represents a heterogeneous mosaic of cultivated fields (resource patches with very short-statured, attractive green crops) and ploughed, bare soil fields with essentially no food resources. In such landscapes, roe deer tend to avoid buildings, roads, and valley bottoms [31].

We predicted that groups would reduce their speed on attractive food patches, such as when they were moving on cultivated fields compared to ploughed, bare soil fields. If roe deer perceive human activity as a potential danger, we would expect that proximity to roads would limit their diffusion rate, particularly if roads play a boundary role. Large groups were expected to move more slowly and have a lower diffusion coefficient than small ones because of the greater inertia generated by group members in the former. Finally, groups were expected to have a smaller diffusion coefficient when they moved in attractive food patches compared to the coefficient observed in ploughed, bare soil fields.

## Methods

### Ethics Statement

This study is restricted to behavioural observations of roe deer and, therefore, excludes any animal handling or invasive experiments. The study thus adheres to the “Guidelines for the Use of Animals in Research”, and to the legal requirements of the country in which the work has been carried out. We obtained permission from all farmers before conducting field observations on their lands.

### Study area and animals

The study was conducted during winter near Machault (45°25′N; 4°30′E) in northeastern France. Groups of roe deer were observed at two study sites, which were separated by a distance of 4 km, with each site consisting of an aggregation of large cultivated fields without hedges.

**Figure 1:**
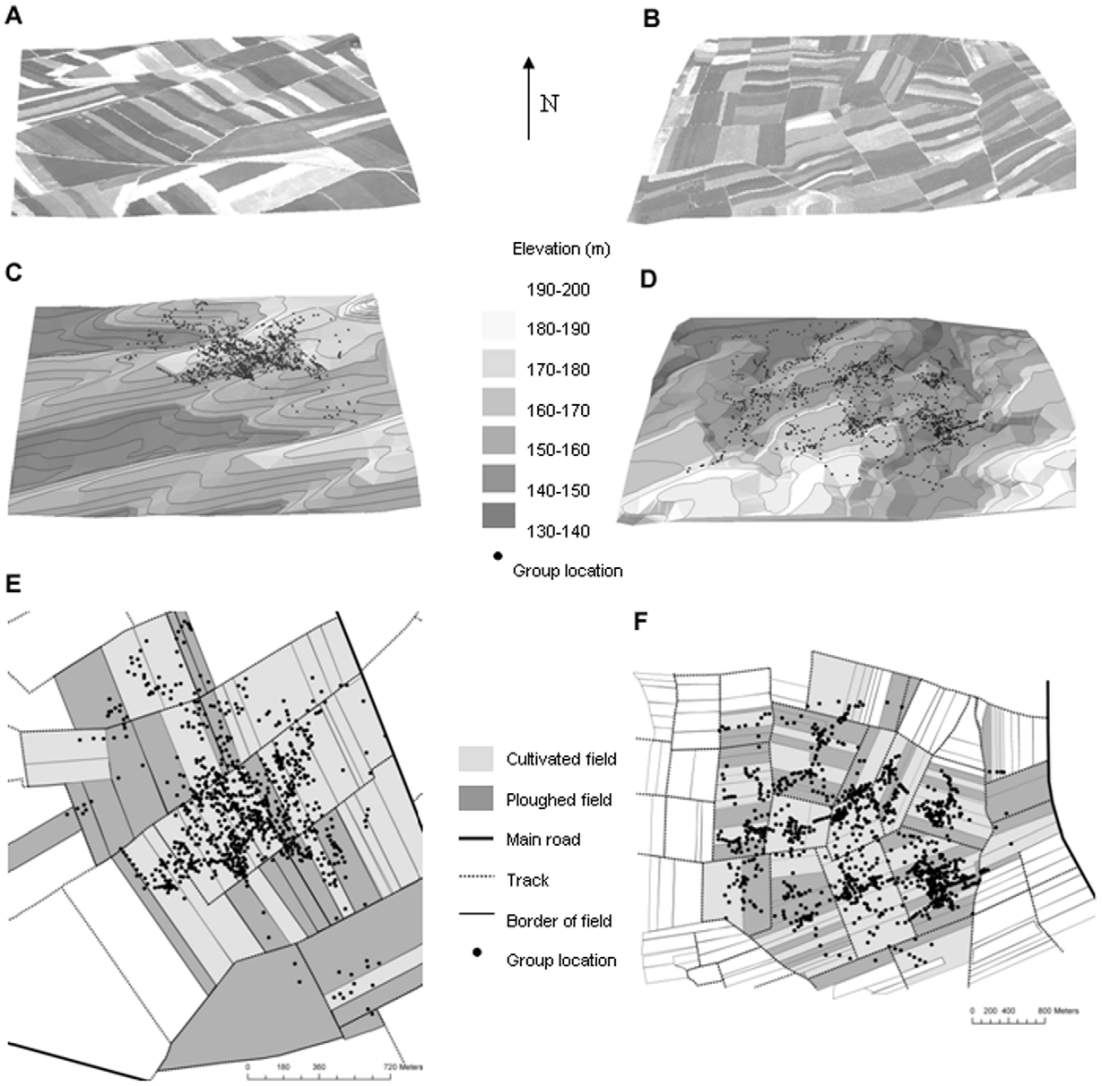
Landscape features of the two study areas. 3D aerial photographs (A, B), field numerical model (C, D), and farming lands (E, F) are presented including all locations of roe deer groups.

We used ARC GIS (Environmental Systems Research Institute, Redlands, CA, USA) to build a numerical field model from 1∶12500 aerial photographs, which had been taken by the French National Geographical Institute to perform a numerical terrain model (Fig. 1a, b). We used the numerical terrain model to identify landscape features at all group locations; these features included elevation, main roads (i.e., departmental roads with heavy traffic), and tracks (i.e., borders of fields, occasionally used by farmers for travel). We also determined if each group location coincided with a cultivated field (i.e., food patch) with very short crops of wheat (*Triticum* spp.), alfalfa (*Medicago sativa*) or sugar beet (*Beta vulgaris*) versus a ploughed field (i.e., foodless patch) characterised by bare soil.

The roe deer population had an estimated density of ca. 3 individuals/km^2^, as estimated from road census during the data collection. As is usually reported for European roe deer, group size was greatest in winter, when adult males are not territorial and the young of the year are fully mobile [49]. Winter groupings were larger than generally reported in forested landscapes, as is typically the case in open cultivated plains [50]–[52].

### Data collection

Fieldwork was conducted from December 2002 to February 2003, and from December 2003 to mid-March 2004, when the low crops provided excellent visibility. Data were collected by the senior author (OP) from both visual observations and video recordings that were conducted from a four-wheel-drive vehicle located at a vantage point more than 200 m from the animals.

To record the movements of roe deer groups in this fusion-fission society, we needed to account for group dynamics. The method that was used has been fully described by Pays et al. [48]; hence, we have only outlined some crucial points. Although animals were not individually marked, determining their fate remained unambiguous during successive fusion and fission events over a complete day of observations due to a combination of continuous visual observations and video-recording. Starting in early morning, a focal group was continuously video-recorded while insuring that all of its members were in the camera's field of view. When this focal group merged with another, the resulting group received a unique identification number and, in turn, was continuously video-recorded until the next event. In the case of group fission, one of the resulting groups was randomly chosen and continuously video-recorded, and so on. Concurrent to video recording, the beginning and ending events of all of the other groups in the observer's field of view (but outside the camera's field) were noted. With this method, we were able to determine each time a group reunited (i.e., again consisted of all and only its original members) during the day, and reassigned it its original identification number with an indicator of recurrence during the day. In the end, every group was assigned a unique identification number, and we considered two groups as different when they varied by at least one individual. For instance, we considered that there were three different groups, either when one initial group split into two resulting groups or when two initial groups merged to form a larger one. The centre of mass of each group (video-recorded or visually monitored) was plotted every 5 min (or every 1 min when the group was splitting up or merging with another group) on 1∶12500 aerial photographs. The high-quality photographs and the geometric shapes of the fields allowed the locations of the groups to be determined very accurately. Centre of mass in large groups was the location of the individual at the most central position within the group and in small groups an average visual estimation of the gravity centre [48]. Because individuals were mostly clumped in the landscape, groups were easy to follow over time and to place on detailed aerial photographs.

Defining whether individuals do or do not form a (spatial) group has been extensively discussed. Ambiguities arise when animals are not tightly clumped [53], in particular if the rate at which individuals join and leave the cluster of group members is high [54]. We encountered no difficulties in distinguishing groups that were simultaneously visible because roe deer are highly clumped in the landscape. Studies investigating group cohesion in roe deer living in an open environment [55] identified that two groups approaching one another could be considered as having merged as soon as their nearest individuals were less than 50 m apart; conversely, an elongating group was considered as having split into two groups as soon as it was divided by a gap greater than 50 m. During the course of the study, we identified 393 roe deer groups, and recorded group size (1–17 individuals) and group lifetime for every group, in a total of 460 hours of field observations.

### Data Analysis

#### Landscapes features

Landscape features included two study sites (Fig. 1A, B) and types of field (cultivated or ploughed), as well as distance to the nearest main road, track (Fig. 1E, F), forest, and crest line (Fig. 1C, D). We ran a principal components analysis (PCA) on the distance between group location and the nearest main road, track, forest, and crest line to reduce the dimensions of the data set. We used scores on the first component (PC1) as an indicator of the distance between group locations and landscape features. In addition, we considered type of field (i.e., ploughed *versus* cultivated) to test whether potential foraging patches encountered along the group's path affected mean group speed and then rate of group diffusion.

#### Group speed

For each group, we calculated travel speed as the distance covered by the group between two successive locations, divided by travel time (i.e., 1 min or 5 min, as indicated above). We then tested the effect of group size (log-transformed), landscape features (PC1 and type of field) and the interactions between these variables on group speed (N groups = 393). On average (± SE), each group was relocated 5.77±0.23 times. We accounted for dependence between the different speed values recorded for a given group by considering group identity as a random factor in a linear mixed effects model with Gaussian distribution. To fulfil normality and homoscedasticity criteria, group speed was log-transformed. Because of the relationship between group size and group lifetime, with larger groups having shorter lifetimes (F_2,510_ = 26.10, P<0.004), we only considered group size in further analyses. Multicollinearity was limited in all models and statistical inferences were valid, as variance inflation factors were consistently less than 10 [56]. We also controlled for the presence of serial autocorrelation using a partial autocorrelation function of the residuals generated by the model [57]. Finally, we verified the dispersion and normality assumptions based on plots of residuals vs. fitted values and on normal quantile-quantile plots of the residuals generated by the models [57]. This analysis was performed using R 2.10.1 (R Development Core Team, Vienna, Austria) including the packages car for the variance inflation function and nlme for the linear mixed effects models.

#### Rate of group diffusion

We also investigated the rate of diffusion by deer groups, with the diffusion coefficient corresponding to 0.25× the slope of the linear relationship between net-squared displacement and time [40], [58] for the deer population. The diffusion coefficient should indicate the diffusion rate expected during the group's lifetime. A steep slope (large coefficient) indicated that groups tended to move quickly away from their initial location. The diffusion coefficient becomes particularly informative over rather long temporal scales, i.e., when the slope becomes independent of time (or number of moves). Therefore, we (1) estimated the diffusion coefficients only for groups that were relocated at least four times (i.e., at least five locations per group), (2) verified that there was no significant effect of the number of locations per group on the diffusion coefficient (linear regression model, F_1–99_ = 0.130, P = 0.719), and (3) included all locations in the same analysis taking into account group identity and (4) considering that, for each group, data were repeated over time (N groups = 157) (i.e., our analysis included group identity as a random factor, with a repeated statement, PROC mixed in SAS 9.2). To investigate whether group size and landscape features alter the diffusion coefficient, i.e., the mean coefficient of the relationship between net-squared displacement and time for the deer population, we included in the mixed-effects model the interaction terms Time×Group size, Time×Percentage of foraging patches encountered along group path, Time×PC1 and Time×Group size×PC1. Notice that covariates other than time only enter the model as part of interaction terms because we were specifically interested in testing their influence on the diffusion coefficient of deer groups. Adding the main effects terms for these covariates would offset the intercept of the relationship between net-squared displacement and time, thereby violating the condition that the group is necessarily at its starting location at time 0. Multicollinearity and dispersion and normality of residuals were also verified, as described in the previous analysis of mean group speed.

## Results

### Group dynamics

Overall 393 groups were observed during the course of this study. Group size averaged 4.9±0.1 (± SE) roe deer. Groups split frequently, but it was also common to observe a group reunite. In fact, after splitting up, 24% of all groups reunited at least once during the day. We found a strong relationship between group size (GS) and the log-transformed group lifetime (GL) (GL = −0.012GS^2^+0.019GS+0.671, F_2–391_ = 26.098, P<0.0001, R^2^ = 0.10). Group lifetime increased until it included up to seven individuals, before decreasing above this threshold. Because of the rather strong relationship, further analyses were based on group size only as the independent variable.

### Landscape features

The first axis (PC1) of the principal component analysis (PCA) explained 52% of the total variation in the distance between group locations and landscape features. According to the loadings (Table 1), PC1 scores increased with distance to the nearest main road and track and the crest line, whereas they decreased with distance to the forest. Mean (± SE) of PC1 scores on site 1 (1.32±0.04) and site 2 (−1.41±0.04) indicated that group locations were mainly farther from main roads and crest lines and closer to forests on site 1 than on site 2 (Mixed-effects Model, F_1,1482_ = 2174.35, P<0.0001). Because of the strong link between PC1 and sites, and to account for spatial variation in distance to landscape features within sites, we restricted our analysis to PC1 scores and did not specifically model site effects. This approach is consistent with the idea that movement decisions should be driven by landscape properties, not by site ID.

**Table 1:** Loadings on PC1 (the first axis explaining 52% of the total variations) of the principal component analysis run on the distance between group locations of roe deer groups and landscape features in an open agricultural landscape in northeastern France.

Factors	Coef
Distance to the nearest main road	0.54
Distance to the nearest track	0.37
Distance to the nearest crest line	0.58
Distance to the nearest forest	−0.49

### Group speed

The speed of roe deer groups was related to both group dynamics and landscape features (Table 2). First, speed decreased with group size. Second, groups tended to move faster on ploughed fields and slower when they encountered food patches along their paths. Third, group speed increased with PC1, which, given the PC1 loadings, implies that groups tended to move faster far from main roads and crest lines and slower near the forest. No interactions significantly improved the model of group speed.

**Table 2:** Factors influencing travel speed (Log-transformed) of roe deer groups in an open agricultural landscape in northeastern France, as assessed using linear mixed-effects models with group ID as a random factor.

Factors	Coef	SE	DF_num_	DF_den_	F	P
Intercept	1.37	0.04	1	1551	4395.33	<0.0001
Log(Group size)	−0.37	0.06	1	115	32.36	<0.0001
Type of field*						
Ploughed	0.03	0.07	1	1551	3.66	0.05
PC1ϕ	0.05	0.03	1	1551	8.42	0.004
Log(Group size)×Type of field	0.02	0.10	1	1551	0.07	0.80
Log(Group size)×PC1ϕ	−0.02	0.04	1	1551	0.40	0.53
Type of field×PC1ϕ	0.01	0.02	1	1551	0.02	0.88

ϕ PC1 scores covary positively with the distance to the nearest road and to the crest line, and negatively with the distance to the forest.

*cultivated field were included as a reference condition.

Pseudo-R^2^ = 0.45.

### Diffusion coefficient

**Figure 2:**
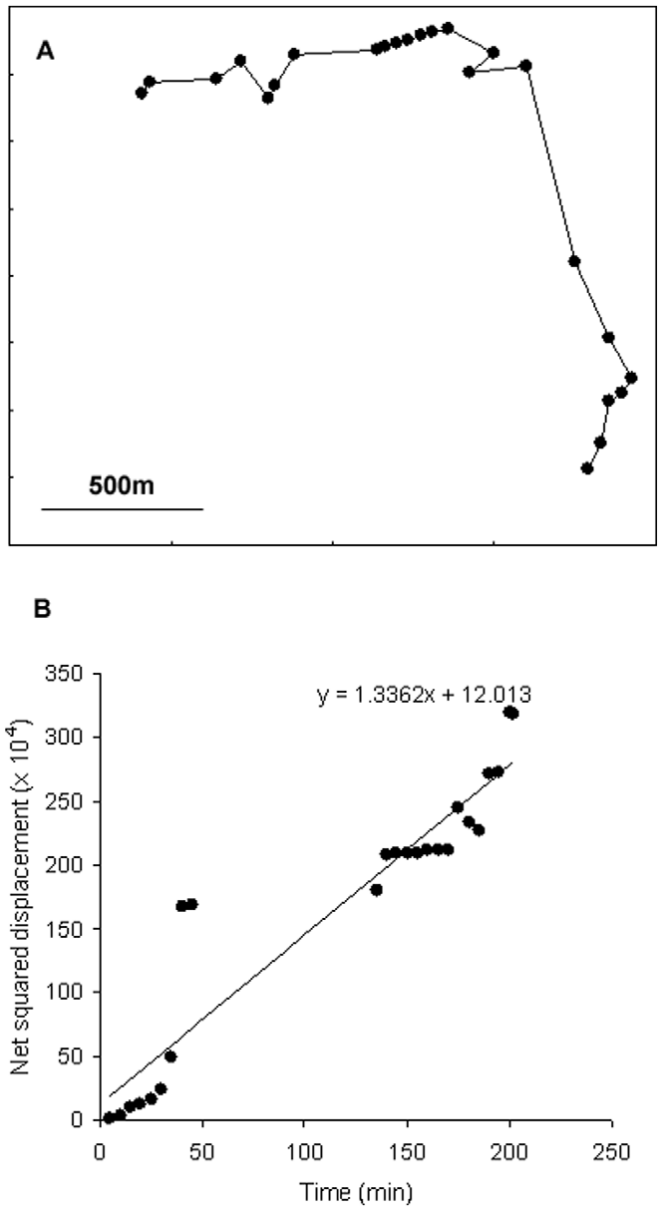
Movement of a roe deer group across the landscape. Movement trajectory (A) and diffusion rate (B) are presented. Diffusion rate is given by the slope between the net-squared displacement (m^2^) and time (min). Time between two consecutive plotted locations is 5 minutes.

**Figure 3:**
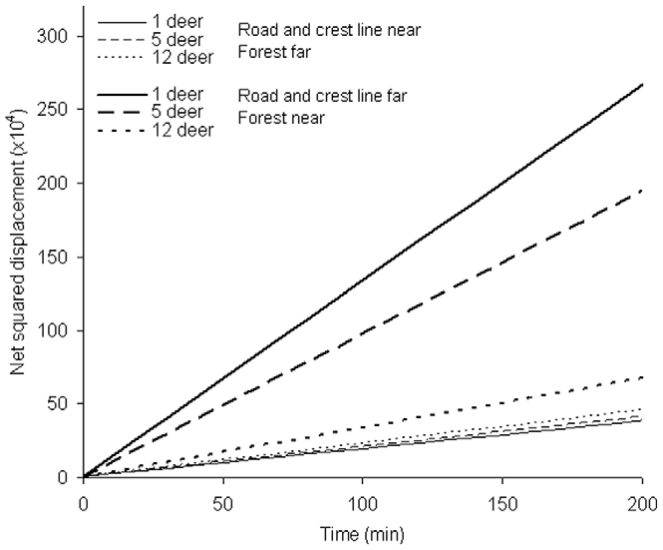
Effect of group size and landscape features on the relationship between net squared displacement and time. See Table 3 for statistical details.

Groups of roe deer displayed broad variations in movement behaviour (Fig. 2). For example, groups increased constantly their distance from their starting location over time (Fig. 2A), leading to a steep slope between net-squared displacement and time (Fig. 2B). Over the 157 groups presenting at least five locations, net-squared displacement increased with time, which corresponded to a positive diffusion coefficient (Table 3). The interaction terms revealed that both group dynamics and landscape features influenced group diffusion rate (Table 3). Deer groups tended (marginally significant, with P = 0.06) to diffuse at a faster rate when they travelled in areas composed of a smaller percentage of potential foraging patches. Groups increased their diffusion rate even further when located near the forest and far from main roads and crest lines (Table 3, Fig. 3). The effect of group size on the diffusion rate depended on the response of groups to landscape features (Time×Group size×PC1). When groups experienced high diffusion rates when travelling near forests and away from main roads and crest lines (i.e., high PC1 scores), small groups had a much faster diffusion rate than large groups (Table 3, Fig. 3). In contrast, when groups experienced low diffusion rates when travelling near roads and crest lines and away from forests (i.e., low PC1 scores), group-size effects on the diffusion rate essentially vanished (Fig. 3). We verified this model prediction (Table 3) by restricting the analysis to the situation where PC1 scores <0 and, as predicted, we found no group size effect on this portion of the dataset (Time×GS, Coef ± SE: −1.94±98.49, F_1,704_ = 0.01, P = 0.98).

**Table 3:** Factors influencing the strength of the diffusion coefficient (i.e., relationship between net squared displacement and time) for groups of roe deer in an open agricultural landscape, northeastern France, as assessed with linear mixed-effects models for repeated observations.

Factors	Coef	SE	F_1,1477_	P
Intercept	8674	26854	0.32	0.75
Time	8035	934	74.02	<0.0001
Time×Percentage of foraging patches	−1554	829	3.52	0.06
Time×Group size	−435	129	11.45	<0.0001
Time×PC1ϕ	5362	627	73.14	<0.0001
Time×Group size×PC1ϕ	−407	91	20.03	<0.0001

ϕ PC1 scores covary positively with the distance to the nearest road and to the crest line, and negatively with the distance to the forest.

Pseudo-R^2^ = 0.36.

## Discussion

Our results show that both landscape components and group features affect the movements of roe deer herds. This finding underscores some constraints that group living can impose on space use patterns and on the rate at which individuals can explore their environment. These constraints should influence the adaptive evolution of group fusion-fission dynamics in ungulate societies.

Our study supports the idea that the spatio-temporal arrangement of habitat attributes plays a key role in determining patterns of movement and habitat use by animals [59]–[62]. We found that roe deer groups decrease their diffusion rate as the percentage of foraging patches increased along their paths, a behaviour consistent with principles of area-restricted search [e.g.], [ 36], [63,64]. When adopting this tactic, individuals exhibit two distinct search modes in relation to food distribution [36]: individuals move slowly with highly sinuous paths during the intensive search mode, whereas they leave an area offering poor quality food by traveling rapidly along rather straight paths during the extensive search mode. Deer groups also tended to travel more rapidly and diffuse at a faster rate when they were far from main roads and crest lines and more slowly when near forests. Proximity to roads thus impacts the movement of groups and, as a result, the speed at which roe deer can find new food patches. The effect of roads on habitat accessibility and spatial patterns of resource use has been reported in many species, including elk *Cervus elaphus* [65], woodland caribou *Rangifer tarandus caribou* [66], plains bison *Bison bison* [67], moose *Alces alces* [68], grizzly bear *Ursus arctos* [69], and grey wolf *Canis lupus* [70], [71].

Besides the influence of landscape attributes on roe deer movements, our study provides new insights into the role of sociality (through group dynamics) on movement behaviour. One of our main findings is that small groups of roe deer can achieve faster diffusion rates than large groups. An increase in group size can thus constrain the speed at which a group can explore its environment while also maintaining its cohesion. However, group size effects on the diffusion coefficient depended on the diffusion rate in the first place. Indeed, large groups only diffused at a slower rate than small groups when moving in areas where local landscape features promote fast diffusion (i.e., in our case, near the forest and away from main roads and crest lines, Fig. 3). Therefore, the upper limit to the diffusion rate might be lower for large than small groups, with the consequence that social constraints on diffusion rate would become detectable sooner for larger groups. A larger number of group members implies greater potential for conflicts in movement directions among individuals, which ultimately constrains large groups to be more static than small groups.

Our study shows that group members are faced with a trade-off dilemma: having a fast exploration rate or gaining social information over a broad area. As they become part of a larger group, they slow down their exploration capacity (e.g. estimated net displacement in 60 min: 932 m for a single individual versus 465 m for a group of 12 roe deer, assuming PC1 = 1.36, Fig. 1), thereby reducing the speed at which they can find suitable food patches over large spatial extents. However, larger groups occupy relatively more space, so that some members are likely to find high-quality patches if they occur within the relatively small area they collectively occupy. Foraging gains from social information provided by more individuals [4], [6] could then somewhat compensate for foraging costs imposed by the slower diffusion rate experienced by large groups. If we further consider that inter-individual competition for food access becomes stronger as group size increases, there comes a point where it would become profitable for a foraging individual to speed up its exploration by leaving a large group and joining a smaller one. The greater stability that we observed for groups comprised of 5–10 roe deer (Fig. 3) should reflect the balance between the benefits and costs of living in these large, open agricultural fields with patchy food distributions, including the impediment to broad-scale food search while remaining in larger groups. The extent to which such scale-dependent cost-benefit trade-offs control the dynamics of group fusion-fission remains an open question.

The slower speed and diffusion rate and, hence, lower potential for broad-scale exploration that we observed in larger groups have important ecological implications. For example, large groups of bison have a longer residency time in meadows than do small groups [72]. The current explanation is that large groups were less at risk and, therefore, may not need to remain as elusive to wolves as small groups by moving constantly. The constraints imposed by group cohesion on movement now provide an additional mechanism that can explain group-size differences in terms of time allocation: large groups are more static than small groups; hence, they have a longer residency time in habitat patches.

A second implication of being in a “slow moving” large group rather than in a “fast moving” small one is that high-quality patches should be encountered at a slower rate. As a corollary, optimal foraging principles would predict broader diets for members of large than small groups as a direct consequence of the slower encounter rate with highly profitable foraging stations [73]–[75]. Accordingly, diet breadth tends to increase with bison group size during summer [6]. In red deer (*Cervus elaphus*), selection by animals for high-quality plants weakens with competitor density [76]. Similarly, feral donkeys (*Equus asinus*) include lower-quality items in their diet as the abundance of conspecifics increases [77]. At a broader scale, eastern grey kangaroos (*Macropus giganteus*) make greater use of low-quality habitats as population density increases [78]. The common explanation for this response is that interference and exploitative competition force individuals to consume less profitable plant species. While this hypothesis remains valid, our study provides an alternative explanation. The constraints imposed by group cohesion on movement should reduce the encounter rate with high-quality food items, leading to diet expansion, especially for members of relatively large groups.

While previous studies have reported that dispersion of deer was affected by habitat fragmentation [79], [80], the formation of large groups of roe deer that we observed in this agricultural landscape during winter could be explained by high inter-individual tolerance outside the rut, habitat openness, and food density. The transition between open and close areas can increase the risk of group fission, as has been reported for bison moving between meadow and forest patches [72]. Roe deer living in the study area do not have to make such transitions because the sites are entirely open. Also, larger groups should generally suffer from greater interference competition [16] and, therefore, they should be more likely to split up relative to small groups when food is limited. However, the cultivated fields are large and offer attractive, very large homogeneous patches, a situation that might contribute to the maintenance of such large groups. Indeed, many fish [81], birds [82], [83], and mammals [84] become non-aggressive when food patches are large [see resource defence theory], [ 85,86].

To summarise, our results highlight the importance of considering group dynamics in predicting the movements of individuals in group fusion-fission societies. Behavioural decisions underlying animal movements and habitat use in large herbivores seem to be strongly guided by factors affecting herd features, such as group size and lifetime. Moreover, how the quality of social relationships and the energetic needs of each individual influence animal movements and fission decisions should stimulate further studies [87]. The strong effect of group size on movement speed and diffusion rate yields additional questions, such as: do unisex (females or males) versus mixed-sex groups explore the environment at the same rate? There are a number of hypotheses and mechanisms related to sexual segregation [88]–[90] that should be tested in regard to the constraints imposed by grouping, which presumably vary with group typology.
